# Accelerated Wound Healing Using a Novel Far-Infrared Ceramic Blanket

**DOI:** 10.3390/life11090878

**Published:** 2021-08-26

**Authors:** Frederick Robert Carrick, Luis Sebastian Alexis Valerio, Maxine N. Gonzalez-Vega, David Engel, Kiminobu Sugaya

**Affiliations:** 1 College of Medicine, University of Central Florida, Orlando, FL 32816, USA; ksugaya@ucf.edu; 2 Burnett School of Biomedical Science, University of Central Florida, Orlando, FL 32816, USA; newmanboy45@icloud.com (L.S.A.V.); Maxine.Gonzalez-Vega@ucf.edu (M.N.G.-V.); David.Engel@ucf.edu (D.E.); 3 MGH Institute for Health Professions, Boston, MA 02129, USA; 4 Centre for Mental Health Research in Association with University of Cambridge, Cambridge CB2 1TN, UK; 5 Carrick Institute, Cape Canaveral, FL 32920, USA; 6 Institute for Scientific Research and Technology Services (INDICASAT), Panama City 0801, Panama; 7 Department of Biotechnology, Acharya Nagarjuna University, Guntur 522510, India

**Keywords:** ceramic, far field infrared, mesenchymal stem cells, regeneration, wound healing, mouse model

## Abstract

Introduction: Wounds are associated with ranges of simple to complex disruption or damage to anatomical structure and function. They are also associated with enormous economic and social costs, increasing yearly, resulting in a severe impact on the wellbeing of individuals and society. Technology that might accelerate wound healing is associated with many benefits to injured people. Methods: BALBc mice underwent symmetrical excisional wounds through the panniculus carnosus. They were divided into a treatment group placed on an autonomous ceramic far-field infrared blanket (cIFRB) and a control group maintained under standard conditions. We also expanded and cultured adipose tissue-derived mesenchymal stem cells (MSCs) on cIFRB and compared them to standard conditions subjected to a scratch injury to compare survival, proliferation, and wound healing. Results: The wound healing of the cIRFB treatment group was significantly faster than the control group of mice. The wound-healing effect of mesenchymal stem cells on cIRFB was also increased and associated with significant migration to the wound area. Conclusions: Wound healing is improved in a mouse model exposed to cFIRB. The ceramic blanket also promotes survival, proliferation, increased migration, and wound healing of MSCs without affecting their survival and proliferation. The utilization of cFIRB in cellular biology and medical applications may be promising in many situations currently explored in animal and human models. This technology needs no direct or battery power source and is entirely autonomous and noninvasive, making its application possible in any environment.

## 1. Introduction

Wounds are associated with ranges of simple to complex disruption or damage to anatomical structure and function [1]. They are also associated with enormous economic and social costs, increasing yearly, resulting in a severe impact on the wellbeing of individuals and society as a whole [2,3,4,5]. The healing of wounds may be prolonged with consequences that affect a cascade of personal and societal functions. For example, impaired healing from many complications is present in nearly 70% of all wounds, resulting in an additional global annual cost of many billions of dollars [4,5,6,7,8]. There must be a significant investment in wound care research and education to address the economic impact on health care challenges associated with wound management [6].

The wound healing phases of hemostasis, inflammation, growth, re-epithelialization, and remodeling represent complex interactive processes that may be impaired with a resultant compromise in successful wound healing [9]. There is a complexity that requires multiple populations of cells to interact with an extracellular matrix and soluble mediators that promote the healing of wounds. Despite this complexity, it is helpful for clinicians to divide the healing of wounds into four distinct phases of coagulation and hemostasis, inflammation, proliferation, and wound remodeling with scar tissue formation [10]. Velnar et al. present a thorough overview of the cellular and molecular mechanisms of wound healing, central to our work [10].

We aimed to decrease the time associated with wound healing and, by association, the complications associated with prolonged healing. In vitro and in vivo wound models have significantly contributed to the current knowledge of wound healing and have formed the basis of understanding cell types and the biological responses that guide therapy development and clinical applications [11]. Novel therapeutic strategies in wound repair are dependent on the development of therapies that might decrease healing times, driving a more significant regenerative phenotype of healing [12]. There are a host of therapies currently utilized that might influence wound healing and impact the complications of prolonged courses of healing. The development of these novel applications should reduce the potential complications associated with wounds [13].

We have taken an interest in treating wounds in military and civilian applications in circumstances where there may not be the immediate availability of a medical facility. We were aware of the innovative use of a non-powered ceramic crystal-induced far-infrared (cFIR) excitation to help hydrocarbon fuels burn in internal combustion engines at a significantly higher efficiency [14]. The wavelength emitted by the cFIR is reported to be associated with therapeutic benefits, including the stimulation of healing, prevention of tissue necrosis, increases of mitochondrial function, improvement of blood flow, and tissue oxygenation, while also acting as an anti-inflammatory agent in a wide range of medical applications [15].

**Figure 1:**
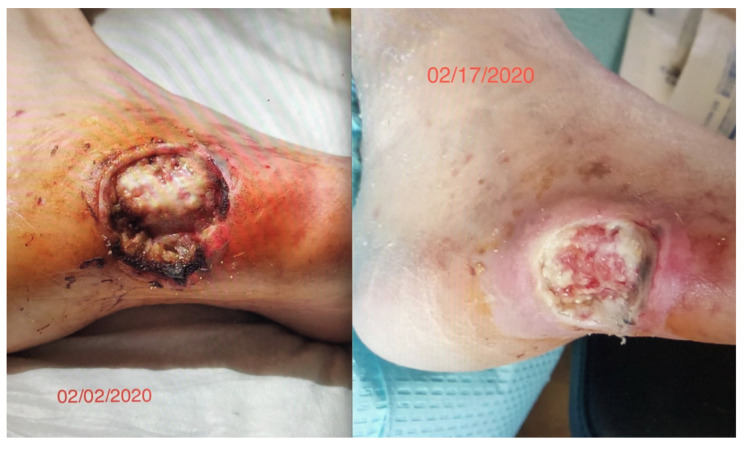
Wound Healing of 8-week persistent lesion over 15 days of exposure to FIR Ceramic Blanket.

We had observed the use of this cIFR technology in a diabetic patient with a worsening non-healing lesion of 8 weeks who was scheduled for an amputation. After a two-week 12 h daily exposure to an embedded ceramic far-field infrared blanket (cIFRB), the wound healing was dramatic. This represented a reversal of a pathological process that we had not seen before (Figure 1).

Consequently, we wanted to investigate the effect of cIFRB in a mouse model excisional wound closure and the proliferation, migration, and cytotoxicity of MSCs in an in vitro wound-healing model. We aimed to measure markers that represented the most important factors in wound healing (adhesion, migration, and vascularization) and chose Fibronectin and CD31 as the best candidates to illustrate this. Fibronectin is a key component in the provisional wound matrix. Its main function during wound healing is in mediating cell adhesion and migration but it also promotes cellular signaling, such as chemotaxis. In normal skin and mucosa, fibronectin is present underneath the basement membrane [16]. CD31 is a standard immuno-stain for evaluating vascular lesions of the skin and as such is considered an important angiogenesis marker that allows measurement of vascularization, another important factor for wound healing [17].

### 1.1. Ethical Approval

All procedures were performed in accordance with the institutional guidelines of the Institutional Animal Care and Use Committee of the University of Central Florida (protocol no. 202000105). The reporting of existing patient data in Figure 1 is exempt from IRB approval because the research involves the collection or study of existing data, documents, records, pathological specimens, or diagnostic specimens that is recorded in such a manner that the subject cannot be identified. The subject gave informed consent consistent with the Declaration of Helsinki for the use of photographs in this investigation.

### 1.2. Biosafety, Biosecurity, and Institutional Safety Procedures

This study was conducted under the biosafety and biosecurity guidelines of the University of Central Florida.

## 2. Materials and Methods

### 2.1. Far Infrared Ceramic Blanket

We used the cIFRB wavelength patented and manufactured for us by Gladiator Therapeutics, LLC. (Emmaus, Pennsylvania). The blankets were composed of ceramic samples made into a shape of 1/3- circumference cutout of a 12-mm long cylindrical tube, with 15-mm I.D. (inner diameter) and 30-mm O.D. (outer diameter). The base mixture of the ceramic FIR-emitting oxides contains, by weight, 20% silicate, 20% alumina, 24% zirconia, and other minority oxides such as sodium monoxide, potassium oxide, ferric oxide, chromic oxide, nickel oxide, and cobalt oxide. The mixture of metal oxides, bonding agents, catalysts, and stabilizers was press-molded to the desired shapes and sintered in a furnace at a temperature above 1100 °C. All ceramic samples were arranged in an array formation secured with silicone rubber (polydimethylsiloxane) mold compound.

### 2.2. Mouse Wound Model

Eight male BALBc mice were housed in standard ventilated cages. The mice were purchased from the Jackson Laboratory, Bar Harbor, ME USA with an age of 8 weeks old when we started the study. All mice were kept on an inverted 12 h light and dark cycle. Ambient temperature (21.0 ± 0.7 °C) and humidity (63 ± 2%) were constant. Water and food were provided to the mice ad libitum before excisional wound surgery. After wound closure rate was observed (day 11), mice were euthanized using CO_2_ asphyxiation followed by cervical dislocation.

### 2.3. Excisional Wound Surgery

Eight BALBc mice were separated 24 h before excisional wounding into individual housing with a removed diet. 1/4th of a 5 g Rimadyl (carprofen) analgesia tablet was placed on the cage floor, and animals were monitored to ensure complete consumption of the tablet.

Adequate anesthesia was induced in an induction chamber with 1–5% isoflurane and an oxygen flow rate of 0.5–1 L/min. The mice were then removed from the chamber and positioned in a nose cone with 1–3% isoflurane and an oxygen flow rate of 0.5–1 L/min to maintain anesthesia. Adequate depth of anesthesia was monitored by respiratory rate, corneal reflex, and response to a toe pinch. Eye lubrication was provided. Hair was removed from the mice dorsum using a hair clipper, followed by applying depilatory cream that sat on the skin for 2 min. The cream was wiped with gauze, and the surgical site was prepared with 50% EtOH and Iodine. The mouse was placed in a lateral recumbent position, and the dorsal skin was folded and raised cranially and caudally at midline using blunt tweezers, forming a sandwiched skinfold. Using a single-use sterile 5 mm diameter circular biopsy punch, full-thickness, symmetrical excisional wounds were created through the panniculus carnosus. The animals were returned to their home cages, half containing the cIFRB, and placed on a heating pad to recover from anesthesia. Animals were monitored and given 1/4th of a 5 g Rimadyl (carprofen) tablet every 12 h post-surgery. The wound was monitored for 11 days, after which time the mice were euthanized. The wound areas were then dissected from the animal’s dorsum and placed in 4% paraformaldehyde for fixation or flash-frozen in liquid nitrogen for molecular analysis. After 24 h, fixed tissue was transferred to a 30% sucrose gradient for cryoprotection and cryostat slicing.

### 2.4. Far Infrared Ceramic Blanket Placement

The cIFRB blankets were placed inside the cages of BALBc mice and became the floor of the cages used in the treatment group of animals. The blankets were exactly manufactured to fit the animal cages and serve as the cage floor for the active group with no blankets in the control group cages. The animals lived on the blankets after surgical wound creation. cIFRB blankets were also manufactured to serve as the floor of the incubator for the active MSC portion of the research with sample dishes placed directly on the blankets.

### 2.5. Wound Closure Analysis

Each animal was assessed every day on days 3, 5, 7, 9, and 11 after excisional surgery until the complete closure of lesions was confirmed. Wound areas were measured using a digital caliper at the major and minor diameters of the lesions. The area of each wound was calculated using the following formula (1):

(*diameter*
*A*/2) × (*diameter*
*B*/2) × π (1)

To calculate the percentage of wound closure, the following formula (2) was used:

(*area of original wound* − *area of actual wound*)/*area of original wound* × 100 (2)

### 2.6. The Antibodies and Reagents

The antibodies used in this study were anti-Fibronectin (rabbit polyclonal 1:400; Novusbio NBP1-91258), and conjugated FITC-CD31 (mouse monoclonal 1:100; Biolegend c.102405). The secondary antibody was Donkey anti-Rabbit Alexa-596 (1:200). Hematoxylin and eosin stain kit (Vectors Laboratory; H-3502).

### 2.7. Immunohistochemistry

Tissue was embedded and frozen in TissueTek OCT compound and sectioned at 20 μm on a cryostat. The tissue was mounted on slides and surrounded by a hydrophobic barrier. The slices were then dried at 37 °C for 30 min and then cooled at room temperature (RT) for 30 min. The tissue was then rehydrated with 1× PBS for 15 min.

IHC-IF: Tissue was then blocked for 30 min in buffer containing 0.5% Triton, 1% BSA, and 10% donkey serum in PBS. Following blocking, slices were washed with 1X PBS twice for 5 min. Slices were incubated overnight at 4 °C in antibody solution at dilutions listed above with 1% donkey serum in PBS. Secondary antibodies were then added and incubated at 2 h at RT. Images were taken using Zeiss 710 with the Zeiss AxioObserver microscope (lens: LD Plan Neofluar 20×/0.4 Ph2 Korr 421351-9970) and analyzed using Image.J.

IHC-H&E: Hematoxylin (Vectors Laboratory; H-3502) was added to the slices to cover the tissue section completely and incubated for 5 min. Slices were rinsed with distilled water (dH_2_O) for 15 s twice. Bluing reagent was added to cover the tissue section completely and incubated for 10–15 s, then washed twice with dH_2_O. Slices were then incubated with 100% ethanol for 10 s. Adequate eosin Y solution was used to cover the tissue section completely and incubated for 2–3 min. Slices were rinsed once again with 100% ethanol for 10 s, then three times for 1–2 min each. Stained tissues were then mounted with coverslips, and images were taken using the microscope and analyzed using ImageJ.

### 2.8. Immunoblotting

Mouse skin biopsies were frozen in liquid nitrogen and macerated with a mortar and pestle for 5 min until the skin samples were completely pulverized. The solid was resuspended at a 1:3 ratio (weight to volume) of ice-cold M-Per lysis buffer (Thermo Scientific, Waltham, MA, USA) with protease, and phosphatase inhibitor cocktail (2 mM 4-Benzenesulfonyl fluoride hydrochloride, 0.3 μM Aprotinin, 130 μm Bestatin, 1 mM EDTA, 14 μm E-64, and 1 μm leupeptin) (Sigma-Aldrich, St. Louis, MO, USA) for 20 min, with intermittent vortex for 5–10 s every 2 min. The supernatant was collected by centrifugation at 16.3 × 10^3^ × g at 4 °C for 10 min, and the insoluble pellet was discarded.

Total protein was measured using the BCA assay (Pierce Thermo Scientific, Waltham, MA USA 02451). From the total cell lysates, 20 μg of total protein (prepared with NuPAGE LDS buffer and reducing agent) was heated at 70 °C for 10 min and cooled on ice for 5 min. The proteins were run on 4–12% NuPAGE SDS-polyacrylamide gel (Invitrogen, Waltham, MA, USA) together with PageRuler Plus pre-stained protein ladder (Thermo Scientific; 26619) and transferred to a nitrocellulose membrane 0.45 μm (Schleicher & Schuell (Keene, NH, USA); Grade BA85). After blocking with 5% non-fat milk in TBST for 1 hr at room temperature (23 °C), the membranes were incubated with specific antibodies overnight at 4 °C. The primary antibodies were from the following sources: CD31 (1:1000, Biolegend (San Diego, CA, USA); 102406), Fibronectin (1:1000, Novus Bio (Littleton, CO, USA); NBP1-91258), and β-Actin (1:1000, Cell Signaling Technology (Danvers, MA, USA), #4970). HRP-conjugated secondary antibodies: Goat anti-Mouse (Invitrogen; #31430) and Goat anti-rabbit (Thermo Scientific; #31460) were used at a 1:5000 dilution.

The antigen-antibody complexes were visualized using the enhanced chemiluminescence West Femto Detection System (Thermo Fisher) as recommended by the manufacturer and visualizing using the ChemiDoc Touch Imaging System. Using ImageJ, the band density (intensity) was determined using the gels configuration and selecting the area of each lane to account for background signaling. Normalized values to Actin were imported to Prism9 to perform the statistical analysis and plots. For determining the Cohen’s coefficient, Jamovi software with esci (exploratory software for confidence intervals) module was used, entering each determined group’s mean and standard deviation.

### 2.9. Stem Cells Culture

Adipose tissue-derived MSCs (Lonza) were expanded in MSC media, which consists of 10% FBS (GIBCO) and 1% Pen/Strep (GIBCO) in DMEM/F12 (GIBCO) using cell culture T75 flask and incubated at 37 °C with 5% CO_2_. When cell density reached more than 80% of confluence, the cells were detached with 0.25% of trypsin-EDTA (GIBCO) for 5 min at 37 °C with 5% CO_2_. We then used a 1:1 ratio of MSC media to neutralize the trypsin activity, following centrifugation at 1200 rpm for 5 min for the precipitation of cells. Finally, we counted the cells for continuing expansion and subculturing according to the assays described below.

### 2.10. MSC Proliferation Assay

For the proliferation assay, the MSCs were sub-cultured at a density of 10,000 cells per well in triplicates, using tissue culture plastic plates of 12 wells plates and stained using the dye BODIPY (Thermo), according to manufacturer conditions. Cells were incubated at 37 °C with 5% CO_2_ using the MSC media described previously. We evaluated proliferation by placing one plate on a standard surface of the incubator and another plate over the ceramic-blanket surface inside the incubator. The experiment was conducted three times, *n* = 3. Three fluorescence and phase contrast photomicrographs for each of the three wells, plated with cells for each surface condition. were taken randomly using an inverted light microscope at days 1–6 for evaluation. Further, we counted the cells manually for each of the micrographs taken along the time points by opening the images with the image J server and evaluated the MSC proliferation, migration, and confluence.

### 2.11. MSC Cell Survival Assay

For the cell survival assay, the MSCs were sub-cultured at a density of 100,000 cells, using tissue culture plastic plates of 12 wells plates with the MSC media. The experiment was conducted three times, *n* = 3. For each experiment, the cells were incubated at 37 °C with 5% CO_2_ by placing one plate on a standard surface of the incubator and another plate over the ceramic blanket’s surface inside the incubator. After 24 h, cells were detached using 0.25% of trypsin-EDTA (GIBCO) for 5 min at 37 °C with 5% CO_2_ and centrifuged to obtain a pellet. The cells were then prepared with the Vibrant Dye Cycle Violet, ready for flow reagent (Invitrogen) according to the manufacturer’s instructions. Cell survival/cell death signals were analyzed by flow cytometry using the FACS Cito Flex S flow cytometer (Beckman) and further analyzed using the FCS express De Novo software (Version 7,Pasadena, CA, USA).

### 2.12. MSC Wound Healing Assay

For the wound healing assay, the MSCs were sub-cultured at a density of 25,000 cells in triplicates, using tissue culture plastic plates of 12 wells plates and incubation at 37 °C with 5% CO_2_ using the MSC media described previously. The control plate was placed on the standard surface of the incubator, and the test plate was placed over the ceramic blanket inside the incubator. After achieving 100% confluency, we stained the live cells using the dye BODIPY (Thermo), according to the manufacturer’s instructions. The experiment was conducted three times, *n* = 3. For each condition, we performed a scratch on each well using the tip of a 1 mL glass pipet, which is 7 mm in diameter. Then, the cells were incubated at 37 °C with 5% CO_2_ until analyzed at different time points. Three fluorescence images and phase-contrast photomicrographs for each of the three wells scratched for each surface condition were taken in the same place at days 1–6 for evaluation using a Zeiss AxioObserver microscope with objectives plan 10× for further analysis with image J.

### 2.13. MSC Analysis

We used Image J to analyze the photomicrograph images. For the fluorescence intensities, each of the fluorescence images was converted into 8 bit pictures. The regions of interest (ROI) were selected at the scratch area for the wound-healing assay before measuring the fluorescence. The program measures the number of pixels regarding the mean gray value from the fluorescence intensity extracted. Samples without the dye were used as the control for autofluorescence, and the mean fluorescence value was calculated from the sample images. The number of cells in the scratched area was also counted manually. For the proliferation assay, we counted the cells manually by opening merged images in image J. We used the FCS express De Novo software for the flow cytometry analysis to extract the counts and percentages of survival. We performed a two-way ANOVA analysis using the GraphPad Prism 7 software (GraphPad Software, San Diego, CA, USA) that estimated the *p* values and calculated the comparisons for statistical analysis. *p* < 0.05 was considered statistically significant and was identified as a single asterisk at the figures edited manually in the same GraphPad Prism 7 software (GraphPad Software, San Diego, CA, USA).

## 3. Results

The healing time of wounds of mice treated with cIFRB was accelerated. The differences in healing times between the treatment and control mice were associated with extremely high statistical and substantive significance (Table 1).

**Figure 2:**
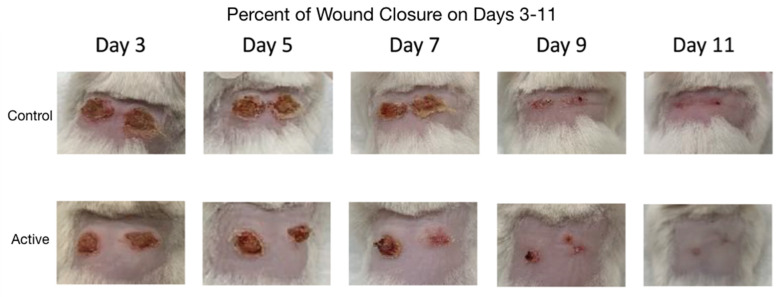
Active cFIRB treated and Control Mice Wound Healing Model.

Photographs comparing the control and active cFIRB mouse’s puncher wound were obtained at the time of all measurements (Figure 2).

**Figure 3:**
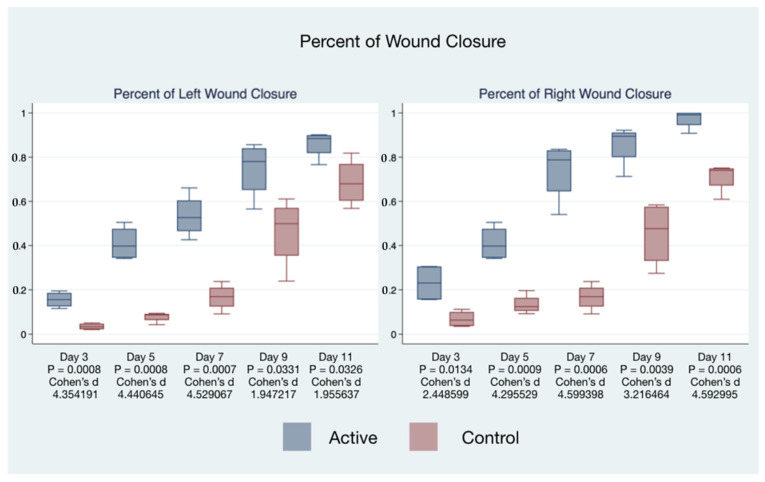
cFIRB treatment accelerates the wound healing process.

**Figure 4:**
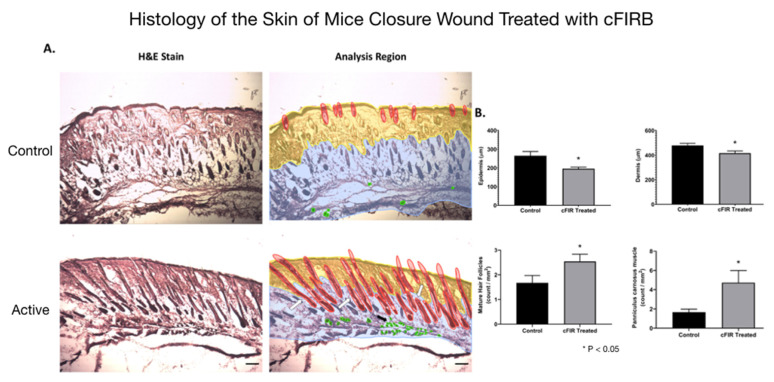
Histology (hematoxylin-eosin (H&E) staining) of the skin of mice closure wound treated with cFIRB. (**A**) Untreated mice versus mice treated with ceramic far-infrared H&E histological staining. The analysis region compares the epidermis (yellow), dermis layers (blue), mature follicles (red), and panniculus carnosus vessels (green) count between cFIRB treated and control mice. (**B**) Mice treated with cFIRB showed a compact epidermis and dermis layer compared to untreated mice at day 13 post-puncher wound, and treated mice had a higher presence of mature hair follicles, fully extending from the dermis to the epidermis, compared to untreated mice. The Panniculus carnosus vessels count was higher for cFIRB treated mice wounds showing the muscle layer’s regeneration, suggesting an efficient wound closure. Each specimen was subjected to H&E staining and photographed at a magnification of 10×. Scale bar = 500 μm. Mature follicles are indicated above the figures with a white arrow and panniculus carnosus vessels by black arrows. Statistical analysis performed *t*-test unpaired with Welch’s correction (assuming different SD values) parametric, and outliers were identified and removed using the ROUT method (Q = 1%), *n* = 8. Significance was considered if *p* < 0.05. *: significance of *p* < 0.05 as depicted in the figure.

The statistical significance and effect sizes of comparisons of the left and right puncher wound closure between active cFIRB and control mice are demonstrated in Figure 3. Histology (hematoxylin-eosin (H&E) staining) of the skin of mice closure wound treated with cFIRB is demonstrated in Figure 4.

**Figure 5:**
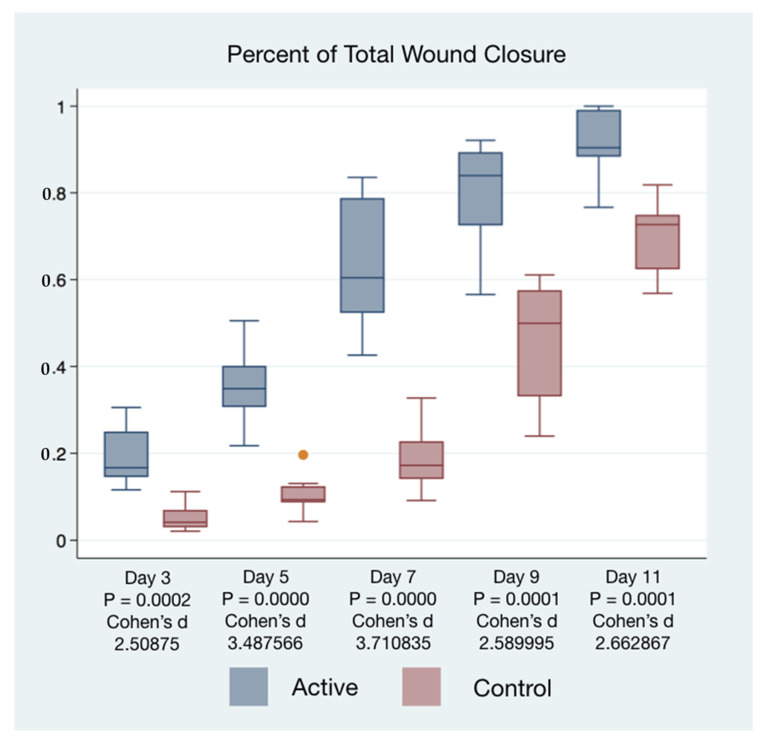
Box Plots of Percentage of Total Wound Closure by Day.

**Figure 6:**
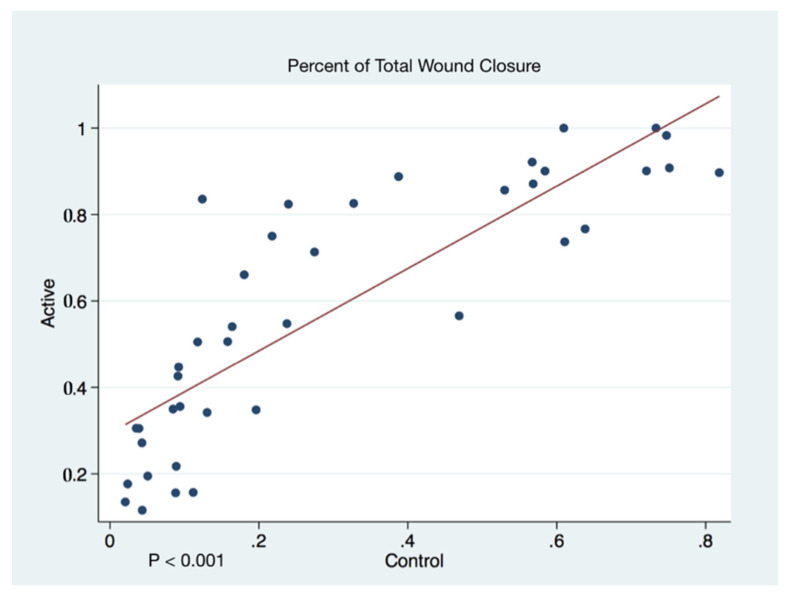
Scatter Plots of Percentage of Total Wound Closure by Day.

Wound closure percentages of the left and right puncher wounds were combined to give a total wound healing percentage for all animals’ closure. The higher rate of wound healing comparing active cFIRB treated and control animals is demonstrated with both statistical and substantive significance in Figure 5 and Figure 6.

**Figure 7:**
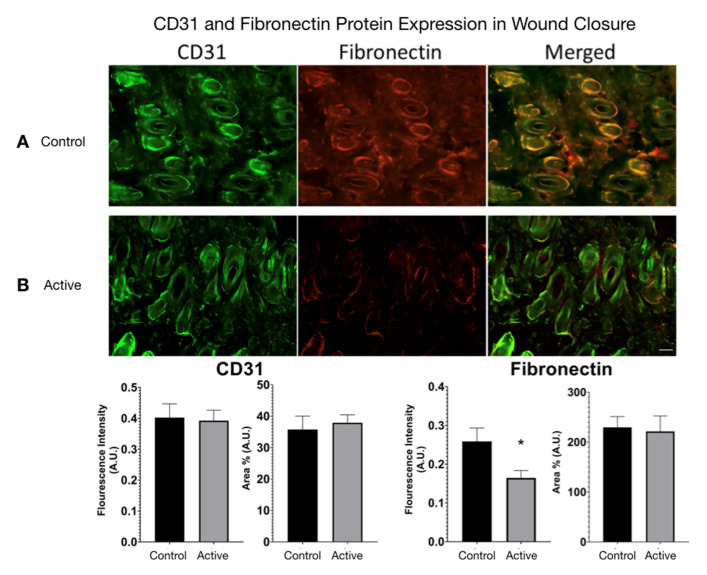
CD31 and Fibronectin Protein Expression in cFIRB treated wound closure mice. (**A**) Untreated mice versus ceramic far infrared treated mice immuno-histological fluorescence staining for CD31, Fibronectin, and DAPI (co-localizes with all nuclei). (**B**) Mice treated with cFIRB showed no significant changes in CD31 protein expression, while treated mice showed a significant decrease in Fibronectin expression. Each specimen was subjected to immunofluorescence staining and photographed at a magnification of 20×. Scale bar = 50 μm. * = *p* < 0.05.

CD31 and Fibronectin Protein Expression were measured in active cFIRB treated and control wound closure mice. (Figure 7) Using ImageJ, color images were converted to 8-bit grayscale. The threshold was generated and manually set to include all fluorescence areas for each filter used. The analysis feature allowed calculation of the area percentage (Area%) for each tissue. Statistical difference was determined by an unpaired *t*-test (two-tailed) with Welch’s correction, and significance was determined if *p*-value < 0.05.

### 3.1. Western Blot

**Figure 8:**
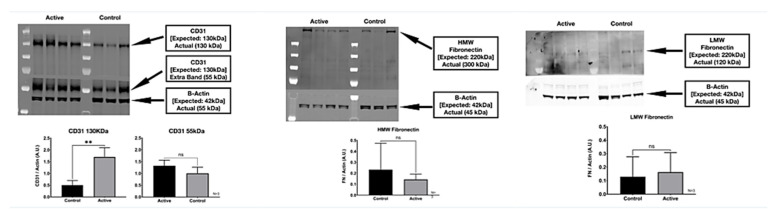
Western blot quantification of cFIR treatment shows significant increases of CD31 protein expression in active vs. controls. Immuno-histological fluorescence shows Fibronectin expression in controls after active has healed. ** *p* < 0.01.

We detected two molecular weight isoforms for Fibronectin that included a high molecular weight (HMW) FN at 300 kDa and a lower molecular weight (LMW) FN at 120 kDa. For Fibronectin, there was no detected difference in protein expression with the treatment. On the other hand, cFIR treatment (active) appears to significantly increase the CD31 (platelet endothelial cell adhesion molecule) protein expression compared to untreated controls. The immuno-histological fluorescence was not as robust as the Western Blot for CD31. It did not demonstrate this difference, whereas it did demonstrate a significant difference in the Fibronectin that the Western Blot did not. (Figure 8)

### 3.2. cFIRB Nontoxic Effects on MSC

To investigate whether the FIR emission from the ceramic blanket has cytotoxic effects, MSCs were cultured with standard serum media over the blanket for 24 h. When cultured under normal serum conditions, the survival of the MSCs has no significant difference when using the standard surface from the incubator and the ceramic pad. Thus, this data suggests that the ceramic-induced FIR blanket has a nontoxic effect on MSCs and would allow survival, proliferation, and wound healing of MSCs.

**Figure 9:**
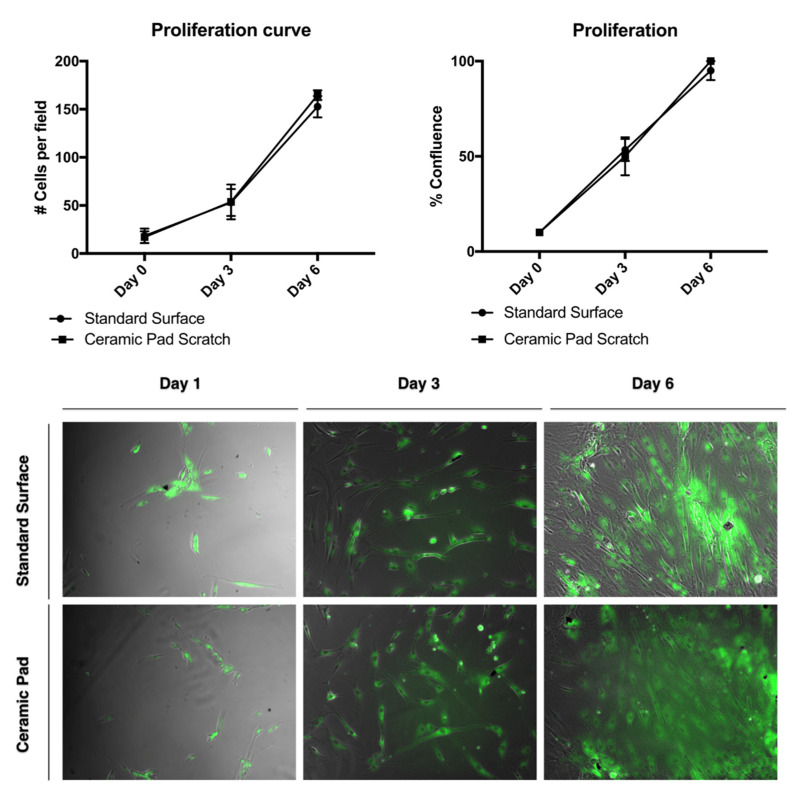
Proliferation of MSCs exposed to the ceramic blanket and standard surface.

Flow cytometry for the cell survival assay results was used to verify the survivability of MSCs after 24 h of exposure to the ceramic blanket using the Vibrant Dye Cycle Violet. To further analyze the effect of the FIR ceramic blanket’s continuous exposure with cell proliferation, the cells were stained with the dye BODIPY and tracked over time. There was no difference in cell proliferation observed, with a doubling rate of about two days (Figure 9)**.** Thus, this data suggests that cFIRB allows the MSCs to proliferate and adhere normally.

**Figure 10:**
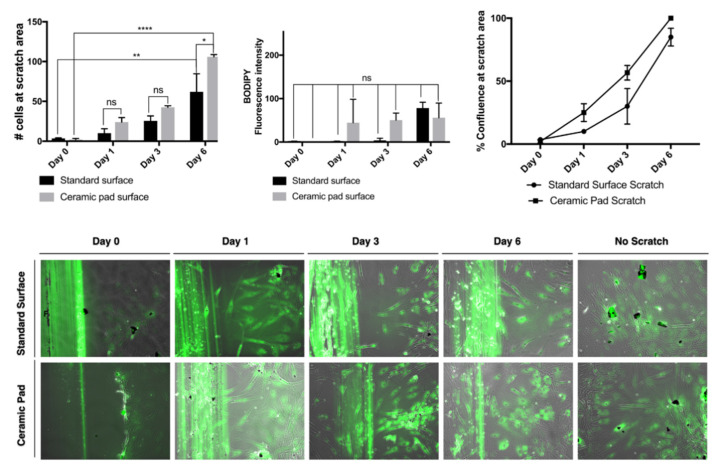
Healing and migration of MSCs exposed to the ceramic blanket and standard surface.* *p* < 0.05 ** *p* < 0.01, **** *p* < 0.001.

The continuous exposure to the ceramic blanket allows the proliferation of the MSCs over time. The doubling rate is about two days, and cells reach 100% confluence simultaneously, as shown in the graph and the merged phase contrast and fluorescent images stained with BODIPY dye. Further, evaluation of whether the continuous exposition to the FIR emission ceramic blanket influenced the cells’ migration was performed by using a scratch assay and staining of the cells using the dye BODIPY to track the cells. If the cells divided, it would be expected that the fluorescence intensities would split as well. However, our data suggest that MSCs exposed continuously to the ceramic blanket can heal a scratch area significantly faster than the control cultured on the incubator’s standard surface (*p* = 0.0021) after six days (Figure 10).

The continuous exposition to the ceramic blanket allows the cells to move faster into the scratched area over time, as shown in the graphs and the merged phase contrast and fluorescent images stained with BODIPY dye. The fluorescence intensities emitted from the BODIPY dye on the cultures did not change significantly, suggesting that the cells did not divide, but did move. The data were analyzed using ANOVA and displayed as S.D. of the mean (*n* = 3). The number of cells that migrated into the scratched area is significantly higher for cells cultured on the ceramic blanket than those cultured on a standard surface (*p* = 0.0147) (Figure 10). The typical fibroblastic-like shape of MSCs cultured on tissue culture-treated plastic plates is conserved in all cells cultured on both the standard surface and the ceramic blanket. Flow cytometry analysis with Violet-ready flow reagent did not differentiate between the cell cultures on the ceramic blanket and the standard surface. Taken together, this data suggests that the MSCs did not receive any significant effect on their survival, adhesion, and proliferation. At the same time, they increased the wound healing effect by increasing their migration to the wound area when cultures were placed over the ceramic blanket emitting FIR.

## 4. Discussion

This study has demonstrated that cFIRB causes wounds to heal faster and may activate the migration signaling of MSCs to the irradiation area to start the regeneration of damaged tissue and accelerate healing, which are robust characteristics of these kinds of cells. Compared to other technologies, such as biomaterials, chemicals or cytokines for increasing wound healing, the ceramic blanket treatment has novel and significant merit. It does not have any toxicity or immune rejection concerns associated with biomaterials since it does not require any material injection. It can be applied to a wound for an extended time without any need for a power supply nor the addition of any agent, which is always needed in the use of chemicals or cytokines to induce migration of the cells.

The FIR ceramic blanket does not require a power supply, and the size is small enough for continuous application to the wounded area [18,19,20,21,22]. Recent studies suggest that FIR can also induce nitric oxide [23] and heat shock proteins [24]. Nitric oxide is the immune system’s molecule to eliminate infection, while heat shock protein is expressed to protect the cells from insults. Thus, the FIR ceramic blanket may increase MSCs migration to the wounded area and may help bodies fight against infection and promote cell survivability in harsh conditions during the wound healing process.

Migration speeds are likely to be enhanced in vitro by stimulating growth factors, enhanced microtubule formation, and moderate increases in cell–cell adhesion. In our study, FIR increased the cell migration without a change in the cells’ survival and proliferation, indicating no influence of growth factor. Although target molecules of FIR in the cell preparation are not exact, they may be related to modulation of the microtubule formation and cell–cell adhesion. Excess cell adhesion can reduce cell migration, and FIR may increase molecular vibration, reducing the stable bindings. FIR action’s possible mechanism may be to activate microtubules related to the migration of the cells, such as F-actin and cell adhesion molecule, to increase migration of the cells.

Mesenchymal stem cells (MSC) are self-renewing multilineage cells that reside in tissue including bone marrow, where they are known to proliferate. When they detect a wound, they migrate out to the bloodstream and migrate to the wound area. Even though MSCs may exist in the skin, their proliferation level there is low and they may be damaged by a wound. Thus migration of MSCs is the most important factor for wound healing, not proliferation or survival 3. cIRFB is also associated with increased kinase activity and actin filament formation which results in an increase the migration. It is evident that MSCs proliferate in the bone marrow and migrate to the wound area via blood flow. The increase in CD31 supports this finding and, in our model, the migrating MSCs are exposed to cIRFB, as also observed in the in vitro experimentation of this study. The consequence of this exposure appears to be related to the MSC increase in actin formation and kinase activity that increases migration into the wound. Once they are situated in the wound, they begin to differentiate into a variety of cell types involving tissue repair [25].

Wu et al. [26] provide insights into the essential and complex molecular machinery underlying polarized stem cell migration that integrate injury-induced migratory signals, remodeling of the cytoskeleton, and polarized cell movements beyond the scope of this report. Microtubule facilitation of cell migration has also been discussed in-depth, explicitly establishing a polarized microtubule array [27]. Microtubule dynamics are controlled partly by spectraplakins that reinforce polarized F-actin and microtubule links central to the polarization of cells sustaining coordinated cell movements [28]. The cellular function dynamics are sustained by rhythmic patterns of oscillation facilitating the cellular microtubular generation of mechanical and electric patterns that impact biomolecular recognition which boosts our inherent ability for self-healing [29]. The use of the cFIRB may affect oscillatory patterns, resulting in the accelerated MSC healing we observed. Further investigations will be needed to confirm this mechanism. However, the enhancement of migration of MSCs could explain the faster healing of tissue damages we have observed in the clinical use of FIR treatments.

## 5. Conclusions

Our findings are novel and have not been investigated or reported previously. Wound healing has been demonstrated to be significantly improved in a mouse model exposed to cFIRB. The ceramic blanket also promotes the survival, proliferation, and wound healing of MSCs. The MSCs did not receive any significant effect on their survival and proliferation. At the same time, they increased the wound healing effect by increasing their migration to the wound area when cultures were placed over the ceramic blanket emitting FIR. The utilization of cFIRB in cellular biology and medical applications may be promising in many situations currently explored in animal and human models. This technology needs no direct or battery power source and is entirely autonomous, making its application possible in many environments. There is increased complexity in treating surface area wounds suffered by military personnel and especially warfighters. The cFIRB accelerates wound healing and may be associated with many benefits to injured personnel who may not have access to electrical or battery-powered devices associated with wound therapy.

## Figures and Tables

**Table 1:** Wound Closure Percentage of Active and Control Mice.

Active Control		Mean	Std Err	Std Dev	95% CI	T	P	Cohen’s d
Day 3	A	0.1932375	0.0258587	0.0731396	0.1320913, 0.2543837	5.0175	**0.0002**	2.508750
	C	0.051575	0.0113343	0.0320582	0.0247736, 0.0783764			
Day 5	A	0.3545875	0.0319625	0.0904036	0.2790082, 0.4301668	6.9751	**0.0000**	3.487566
	C	0.1059500	0.0157815	0.0446369	0.0686326, 0.1432674			
Day 7	A	0.6365875	0.0546517	0.1545785	0.5073567, 0.7658183	7.4217	**0.0000**	3.710835
	C	0.1875750	0.0259511	0.0734009	0.1262103, 0.2489397			
Day 9	A	0.8007750	0.0428342	0.1211535	0.6994881, 0.9020619	5.1800	**0.0001**	2.589995
	C	0.4578500	0.0504769	0.1427701	0.3384912, 0.5772088			
Day 11	A	0.9157875	0.0279039	0.0789241	0.8498053, 0.9817697	5.3257	**0.0001**	2.662867
	C	0.6983250	0.0298104	0.0843165	0.6278346, 0.7688000			

## Presentation

This research was accepted for presentation at the 2021 Military Health System Research Symposium, Kissimmee, Florida, 23–26 August 2021.

## References

[1] Robson M.C., Steed D.L., Franz M.G. (2001). Wound healing: Biologic features and approaches to maximize healing trajectories. Curr. Probl. Surg..

[2] Natarajan S., Williamson D., Stiltz A.J., Harding K. (2000). Advances in wound care and healing technology. Am. J. Clin. Dermatol..

[3] Fearns N., Heller-Murphy S., Kelly J., Harbour J. (2017). Placing the patient at the centre of chronic wound care: A qualitative evidence synthesis. J. Tissue Viability.

[4] Sen C.K., Gordillo G.M., Roy S., Kirsner R., Lambert L., Hunt T.K., Gottrup F., Gurtner G.C., Longaker M.T. (2009). Human skin wounds: A major and snowballing threat to public health and the economy. Wound Repair Regen. Off. Publ. Wound Health Soc. Eur. Tissue Repair Soc..

[5] Sen C.K. (2021). Human Wound and Its Burden: Updated 2020 Compendium of Estimates. Adv. Wound Care.

[6] Sen C.K. (2019). Human Wounds and Its Burden: An Updated Compendium of Estimates. Adv. Wound Care.

[7] Guest J.F., Ayoub N., McIlwraith T., Uchegbu I., Gerrish A., Weidlich D., Vowden K., Vowden P. (2015). Health economic burden that wounds impose on the National Health Service in the UK. BMJ Open.

[8] Ektare V., Khachatryan A., Xue M., Dunne M., Johnson K., Stephens J. (2015). Assessing the economic value of avoiding hospital admissions by shifting the management of gram+ acute bacterial skin and skin-structure infections to an outpatient care setting. J. Med. Econ..

[9] Rodrigues M., Kosaric N., Bonham C.A., Gurtner G.C. (2019). Wound Healing: A Cellular Perspective. Physiol. Rev..

[10] Velnar T., Bailey T., Smrkolj V. (2009). The wound healing process: An overview of the cellular and molecular mechanisms. J. Int. Med. Res..

[11] Boyko T.V., Longaker M.T., Yang G.P. (2017). Laboratory Models for the Study of Normal and Pathologic Wound Healing. Plast. Reconstr. Surg..

[12] Ansell D.M., Holden K.A., Hardman M.J. (2012). Animal models of wound repair: Are they cutting it?. Exp. Dermatol..

[13] Holden J.E. (2011). Putting the bio in biobehavioral: Animal models. West. J. Nurs. Res..

[14] Wey A. Infrared-excitation for Improved Hydrocarbon Fuels Combustion Efficiency–Concept and Demonstration. SAE Technical Papers 2010-01-1953. https://www.sae.org/publications/technical-papers/content/2010-01-1953/.

[15] Walski T., Dabrowska K., Drohomirecka A., Jedruchniewicz N., Trochanowska-Pauk N., Witkiewicz W., Komorowska M. (2019). The effect of red-to-near-infrared (R/NIR) irradiation on inflammatory processes. Int. J. Radiat. Biol..

[16] Patten J., Wang K. (2021). Fibronectin in development and wound healing. Adv. Drug Deliv. Rev..

[17] El-Tookhy O.S., Shamaa A.A., Shehab G.G., Abdallah A.N., Azzam O.M. (2017). Histological Evaluation of Experimentally Induced Critical Size Defect Skin Wounds Using Exosomal Solution of Mesenchymal Stem Cells Derived Microvesicles. Int. J. Stem Cells.

[18] Huang Z., Tian J., Yu B., Xu Y., Feng Q. (2009). A bone-like nano-hydroxyapatite/collagen loaded injectable scaffold. Biomed. Mater. (Bristol Engl.).

[19] Janarthanan G., Kim I.G., Chung E.J., Noh I. (2019). Comparative studies on thin polycaprolactone-tricalcium phosphate composite scaffolds and its interaction with mesenchymal stem cells. Biomater. Res..

[20] Kim H.Y., Yu Y., Oh S.Y., Wang K.K., Kim Y.R., Jung S.C., Kim H.S., Jo I. (2019). Far-Infrared Irradiation Inhibits Adipogenic Differentiation and Stimulates Osteogenic Differentiation of Human Tonsil-Derived Mesenchymal Stem Cells: Role of Protein Phosphatase 2B. Cell. Physiol. Biochem. Int. J. Exp. Cell. Physiol. Biochem. Pharmacol..

[21] Wang X., Ma B., Xue J., Wu J., Chang J., Wu C. (2019). Defective Black Nano-Titania Thermogels for Cutaneous Tumor-Induced Therapy and Healing. Nano Lett..

[22] Zhang Z., Dai Q., Zhang Y., Zhuang H., Wang E., Xu Q., Ma L., Wu C., Huan Z., Guo F. (2020). Design of a Multifunctional Biomaterial Inspired by Ancient Chinese Medicine for Hair Regeneration in Burned Skin. ACS Appl. Mater. Interfaces.

[23] Kim S., Park H.T., Soh S.H., Oh M.W., Shim S., Yoo H.S. (2019). Evaluation of the immunobiological effects of a regenerative far-infrared heating system in pigs. J. Vet. Sci..

[24] Kimura I., Yamamoto T., Nakamura K., Uenishi T., Asai T., Kita M., Kanamura N. (2018). Effects of far infrared radiation by isotropic high-density carbon on the human oral mucosa. Arch. Oral. Biol..

[25] Fu X., Liu G., Halim A., Ju Y., Luo Q., Song A.G. (2019). Mesenchymal Stem Cell Migration and Tissue Repair. Cells.

[26] Wu X., Shen Q.-T., Oristian D.S., Lu C.P., Zheng Q., Wang H.-W., Fuchs E. (2011). Skin stem cells orchestrate directional migration by regulating microtubule-ACF7 connections through GSK3β. Cell.

[27] Watanabe T., Noritake J., Kaibuchi K. (2005). Regulation of microtubules in cell migration. Trends Cell Biol..

[28] Kodama A., Karakesisoglou I., Wong E., Vaezi A., Fuchs E. (2003). ACF7: An essential integrator of microtubule dynamics. Cell.

[29] Facchin F., Canaider S., Tassinari R., Zannini C., Bianconi E., Taglioli V., Olivi E., Cavallini C., Tausel M., Ventura C. (2019). Physical energies to the rescue of damaged tissues. World J. Stem Cells.

